# The prognostic value of cytotoxic T-lymphocyte antigen 4 in cancers: a systematic review and meta-analysis

**DOI:** 10.1038/srep42913

**Published:** 2017-02-17

**Authors:** Pingping Hu, Qiqi Liu, Guodong Deng, Jingxin Zhang, Ning Liang, Jian Xie, Jiandong Zhang

**Affiliations:** 1 Department of Radiation Oncology, Qianfoshan hospital affiliated to Shandong University, 16766 Jingshi Road, Jinan, 250014, P.R. China; 2 Division of Oncology, Department of Graduate, Weifang Medical College, 7166 Baotongxi Road, Weifang, 261053, P.R. China

## Abstract

The outcomes of studies analyzing the prognostic role of CTLA-4 in cancers are controversial. Therefore, the aim of our meta-analysis was to clarify the correlation between CTLA-4 expression and OS in different cancer cases. Relevant literature was searched using PubMed, EMBASE, Web of Science, and the Cochrane Library. The clinicopathological features, hazard ratio (HR) and 95% confidence intervals (CI) were collected from these studies and were analyzed using Stata version 12.0 software. The pooled HR values showed no significant correlation between CTLA-4 expression levels and OS in relation to tumors (HR: 1.24, 95% CI: 0.98–1.56, I2 = 71.7%, P = 0.000). Further subgroup analyses were conducted and categorized by experimental methods, CTLA-4 sources and cancer types. The survey showed a significant correlation (HR: 1.47, 95% CI: 1.14–1.89) between high expression of CTLA-4 and OS in the SNP subgroup, and subgroups analyzing by PCR (HR: 1.50, 95% CI: 1.20–1.86) and flow cytometry (HR: 2.76, 95% CI: 1.49–5.14). In addition, our analysis observed significant differences between patients and controls in inCTLA-4+CD4+ lymphocytes, surCTLA-4+CD4+ lymphocytes, inCTLA-4+CD8+ lymphocytes, and surCTLA-4+CD8+ lymphocytes. Knowledge of the effects of CTLA-4 could potentially be used to effectively guide appropriate prognosis and therapeutic strategies in cancer patients.

Despite recent progress and newly approved drugs, prognosis of advanced stage patients undergoing systemic therapy are still suboptimal and new treatment option is needed. The reactivity of immune system to tumors mainly depends on the activation of immune cells including natural killer cell, macrophage, and T lymphocyte especially. The failure of immune surveillance is a dramatically responsible for the cancer development. Recently, clinical trials have gone far with immunotherapy via checkpoint blockade^1^,^2^. Immune checkpoint was utilized by human body to maintain immune homeostasis and protected normal tissue from being attacked by autoimmune system^3^. Cancer cells have been discovered to escape attack of immune system, as well as break immune homeostasis via the immunosuppressive mechanism^4^. Unlike traditional therapies that exhibiting direct cytotoxic effects, the blockade of immune checkpoint is to enhance the antitumor effect of cytotoxic T cells and against the suppressed immunity in tumors^3^. Quantities of clinical trials and patients who have been reported with durable long-term response have proved the antitumor function via targeting checkpoint pathways. Cytotoxic T-lymphocyte-associated protein 4 (CTLA-4) is one of the most studied hotspots among the checkpoints.

CTLA-4 is an inhibitory molecule expressed on T cell upon activation and plays crucial roles in the balance between the pro- and anti-immune via downregulating T cell signaling^5^,^6^. Human CTLA-4 consist of two isoforms: a membrane-bound receptor isoform (mCTLA-4) with both extracellular and intracellular domains and a secreted, soluble isoform (sCTLA-4), which only have the extracellular domain for ligand-binding^7^. These two isoforms form negative feedback loops to reduce T cell activation both intrinsically and extrinsically, thus to maintain immune self-tolerance and homeostasis. CTLA-4 interacts with B7 ligands (CD80/CD86) expressed on antigen presenting cells to inhibit cell proliferation, cytokines (including interleukin-2 and interferon) production, and cell cycle progression^6^. As a major co-stimulatory receptor highly expressed on the surface of resting T cells, CD28 could be engaged by CTLA-4 to prevent anergy and cell death^8^,^9^. Stimulation of naive T cells through T cell receptors could cause rapid and transient translocation of intracellular CTLA-4 to cell surfaces and/or extracellular secretions^10^. Constitutive CTLA-4 expressions on T regulatory cell (Treg) reduce the level of B7 ligand on antigen presenting cells, further inhibiting effector T cell immunity^11^. In addition, CTLA-4-expressing cells trans-endocytose ligands on neighboring cells, preventing CD28 co-stimulation^12^.

S-CTLA-4 could also interact with B7, inhibiting the activity of T cells via the interfere with CD28 signaling, and blocking soluble CTLA-4 enhances antigen-driven peripheral blood mononuclear cell responses. However, several studies showed that sCTLA-4 also functions in downregulating the negative signal of CTLA-4 in T-cell responses, which indicated that the immunoregulatory functions of sCTLA-4 might be complicated. It has been established and shown that CTLA-4 protein expression appears to be important for tumors to evade host immune surveillance in cancers. However, the clinical implications of CTLA-4 expression in tumors or immune cells in the tumor microenvironment are still controversial, and the potential for CTLA-4 as a prognostic marker has been complicated by differences in study populations and methods. Thus, our meta-analysis aims to illustrate the correlation between CTLA-4 expressions and overall survival (OS), as well as its prognostic role in cancer patients. We also analyzed the percentage of lymphocyte subsets (CD4+, CD8+ and CD19+) expressing surface and/of intracellular CTLA-4 in cancer patients and healthy volunteers, aiming to figure out the correlation between lymphocytes CTLA4 locations and their susceptibility to cancers.

## Materials and Methods

### Literature search

All available published literature concerning the prognostic value of CTLA-4 was systematically searched using electronic database. PubMed, EMBASE, Web of Science, and the Cochrane Library were searched for relevant articles. We also searched for potentially additional studies in references from eligible studies and correlated systematic reviews. The potential eligible literatures were restricted to English publications to facilitate understanding. The search strategy was (CTLA-4 OR cytotoxic T lymphocyte-associated antigen-4) AND (prognosis OR outcome OR survival) AND (cancer OR neoplasm OR carcinoma OR tumor). The final search was performed on July 15, 2016.

### Eligibility criteria

Two investigators (LQ and HP) initially assessed the relevance by checking the titles and abstracts of the articles. The further eligibility criteria for our meta-analyze were as follows: (1) trials was dealing with human cancers, (2) an association between CTLA-4 and OS, or (3) dealing with CTLA-4 protein present on the surface and/or intercellular of lymphocyte, (4) Hazard ratios (HR) could be extracted directly or calculated indirectly in those articles dealing with OS, and (5) the studies represented original articles. Duplicate articles were excluded by checking the title, author name and study detail. We contacted authors for further details when data were not provided in articles. All the disagreements were resolved through discussion.

### Data extraction

Two investigators (LQ and HP) independently conducted the data extraction using a pre-determined form. The following information was collected when analyzing the association between CTLA-4 and OS: the first author, publication year, country, number of included patients, tumor type, stage, source of CTLA-4, detective method, analytic method, cut-off criteria, cut-off value, and survival data (Table 1). When analyzing the sur- and/or in- CTLA4 lymphocytes, we extracted the number of cases and controls, and available genotype frequencies information additionally. Incomplete data was determined by the accrual periods, the median follow-up periods, analysis and submission dates, as described by Tierney *et al*.^13^ All the disagreements were resolved through discussion.

### Statistical analysis

To clarify the correlation between CTLA-4 and clinical data, we calculated pooled estimates of HR which were extracted in eligible articles^13^. Clinical data of patients including tumor histology, stage, smoking history, sex, and age were collected and analyzed in the article. A sensitivity analysis was performed to investigate the heterogeneity for all analyses which can be reflected by P-values and I^2^ values. Random-effect model was applied when there appeared to have heterogeneity between studies (I^2^ > 50% or P < 0.1), otherwise, the fixed-effect model was used. The sources of heterogeneity can be identified by subgroup stratification analyses according to experimental methods, CTLA-4 sources and cancer types. Notably, the analysis can only be performed when more than two articles were included in each subgroup. Publication bias was detected using Begg’s and Egger’s tests^14^,^15^. P < 0.05 was considered statistically significant. All data were analyzed using Stata software (version 12.0).

## Results

### Characteristics of eligible studies

**Figure 1:**
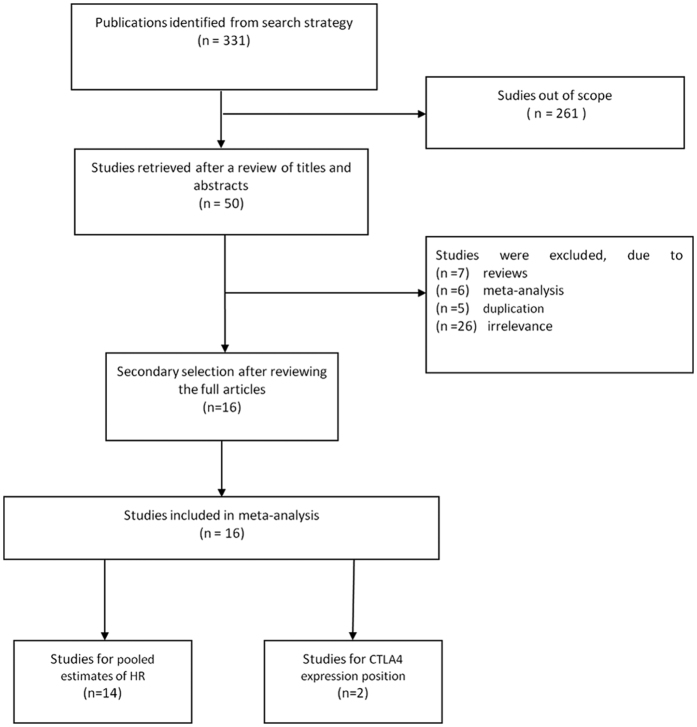
Meta-analysis flow chart.

Our search retrieved 331 articles after initial searching for pooled estimates of HR, which is utilized to explore the correlations between CTLA-4 expression and clinical data of cancer patients. We also searched for studies dealing with sur-CTLA4 and/or intercellular CTLA4 expression of lymphocytes. After glancing the titles and abstracts of the articles, 50 studies were remained whose full text was reviewed. Among the articles, 13 studies were excluded due to editorial, letters, reviews and meta-analysis, 5 articles were excluded due to duplication, 26 articles were excluded due to irrelevance to the prognostic value or expression position of CTLA-4, and/or inadequate data to calculate HRs and CIs. Finally, a total of 14 publications were finally selected for analyzing the prognostic value of CTLA-4, and 2 other studies were relevant to CTLA-4 protein expression position (Fig. 1). Some studies involved several subtypes, so they were analyzed repeatedly. The characteristics of these studies are listed in Table 1.

A total of 2,932 patients were statistically calculated for OS in 14 studies^5^,^7^,^16^,^17^,^18^,^19^,^20^,^21^,^22^,^23^,^24^,^25^,^26^,^27^. The median number of patients in the articles was 151 (range 24–780). Eligible articles consisted of 3 articles for lung cancer, 4 for malignant hematologic diseases, and 7 other tumor types. Five studies were reported for stages I-III, six for stages I-IV, two for II-III and only one for stage III-IV, six more didn’t mention the stage information. In terms of CTLA-4 detection, seven eligible articles used immunohistochemistry (IHC) to assess CTLA-4 expression, six used polymerase chain reaction (PCR), one used a microarray database, and the last one used both ELISA and IHC. Cut-off values were used to divide the expression of CTLA-4 into high and low levels, among which median or mean levels were mostly chosen in these studies. The majority of studies utilized median value as cut-offs, three used a ROC curve and six didn’t mentioned. In addition, in the 2 eligible studies for expression position of CTLA-4 protein in lymphocytes, one study is for lung cancer and the other for laryngeal carcinoma. The following variants were evaluated when analyzing sur-/in- CTLA-4 lymphocytes: sur-/in-CTLA-4 +CD4+ lymphocytes, sur-/in-CTLA-4+CD8+, and sur-/in-CTLA-4+CD19+ lymphocyte. We also conducted subgroup analysis by variants and CTLA-4 protein expression region.

### Publication bias

**Figure 2:**
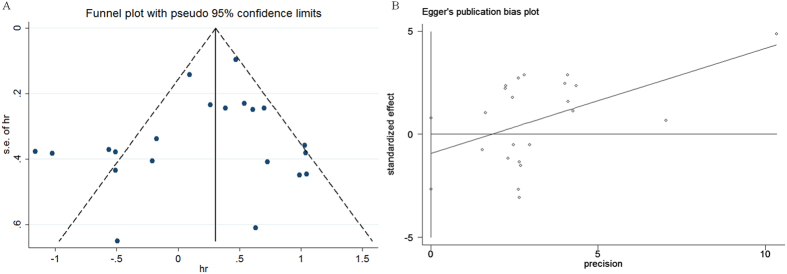
Funnel plots and Egger’s test in the context of OS without and with trim and fill. The pseudo 95% CI is computed as part of the analysis that produces the funnel plot and Egger’s test, and corresponds to the expected 95% CI for a given standard error (SE).

**Figure 3:**
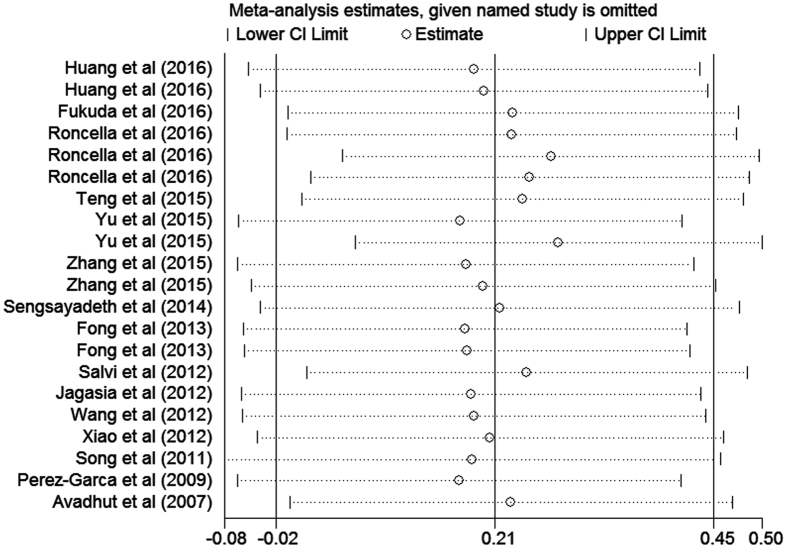
Sensitivity analysis of OS in the meta-analysis.

Egger’s and Begg’s tests were examined to detect publication bias for our article. Of the 14 studies correlated with OS information, the p value was 0.277 for Egger’s test and 0.651 for Begg’s test, which suggested that this study had no publication bias when evaluating OS for CTLA-4 (Fig. 2). In addition, we evaluated the influence of each study on the overall meta-analysis estimate by sensitivity analysis, which indicated that all data correlated with prognostic role of CTLA-4 expressions in all cancer patients were stable with OS as the endpoint in our analysis (Fig. 3).

### CTLA-4 expression and OS

**Figure 4:**
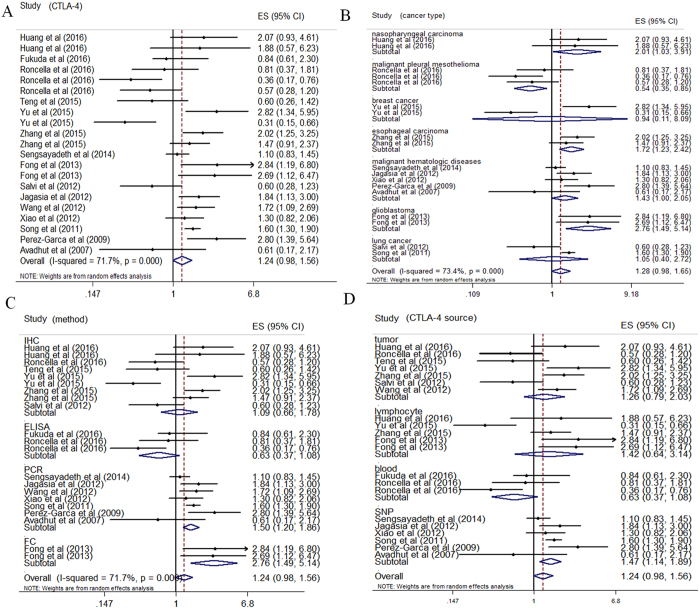
Forrest plots evaluating maximally adjusted association between CTLA-4 expression and OS. (**A**) Forrest plot to assess the overall effect of CTLA-4 on OS in all cancer patients. (**B**) Forrest plot to assess the effect of CTLA-4 on OS in subgroups divided by cancer types. (**C**) Forrest plot to assess the effect of CTLA-4 on OS in subgroups divided by experimental methods. (**D**) Forrest plot to assess the effect of CTLA-4 on OS in subgroups divided by CTLA-4 sources.

The pooled HR values showed no significant correlation between CTLA-4 levels and OS in tumor patients (HR: 1.24, 95% CI: 0.98–1.56) (Fig. 4). In addition, significant heterogeneity (P = 0.000, I^2^ = 71.7%) was observed and thus random-effect models were performed. Subsequently, the examined studies were categorized into subgroups, including cancer types, CTLA-4 sources, and experimental methods, to minimize heterogeneity and to further investigate the prognostic role of CTLA-4. The survey showed a significant correlation (HR: 1.47, 95% CI: 1.14–1.89) between high expression of CTLA-4 and OS in the subgroup of single nucleotide polymorphisms (SNPs). Besides, CTLA-4 was indicated to be a good prognostic indicator for the studies in the subgroups analyzing by PCR (HR: 1.50, 95% CI: 1.20–1.86) and/or flow cytometry (HR: 2.76, 95% CI: 1.49–5.14). In addition, the subgroup analyses performed according to cancer types indicated that in nasopharyngeal carcinoma (HR: 2.01, 95% CI: 1.03–3.91), esophageal carcinoma (HR: 1.72, 95% CI: 1.23–2.42), glioblastoma (HR: 2.76, 95% CI: 1.49–5.14), and malignant hematologic diseases (HR: 1.43, 95% CI: 1.00–2.05), CTLA-4 was a good prognostic indicator, while CTLA-4 was associated with poor outcome in malignant pleural mesothelioma (HR: 0.54, 95% CI: 0.35–0.85). We found that heterogeneity declined dramatically in a majority of subgroups including nasopharyngeal carcinoma, malignant pleural mesothelioma, esophageal carcinoma, malignant hematologic diseases, and glioblastoma. However, we didn’t observe any significant in the IHC subgroup (HR: 1.09, 95% CI: 0.66–1.78), as well as cancer subgroup (HR: 1.26, 95% CI: 0.97–2.03) and lymphocyte subgroup divided according to CTLA-4 sources (HR: 1.42, 95% CI: 0.64–3.14). Notably, this analysis indicated that sCTLA-4 subgroup, analyzing by ELISA, has a strong tendency to be correlated with good prognosis in cancers, though no significance was showed in our article (HR: 0.63, 95% CI: 0.37–1.08). Notably, Yu *et al*.^7^ indicated that the expression of HER2 was not correlated with the density of interstitial CTLA-4+ lymphocytes in breast cancer patients, while HER2 was independent predictors of shorter OS and disease-free survival (DFS).

### Percentage of CTLA4+ lymphocyte subsets

Our analysis observed increased expression of sur- and in-CTLA-4 of lymphocytes in majority of investigated patients, as well as significant differences between patients and controls in inCTLA-4+CD4+ lymphocytes (8.66 ± 7.49 and 4.17 ± 6.11 respectively, P = 0.002), surCTLA-4 +CD4+ lymphocytes (0.78 ± 0.65 and 0.54 ± 0.51 respectively, P = 0.000), inCTLA-4+CD8+ lymphocytes (9.12 ± 9.72 and 3.5 ± 7.65 respectively, P = 0.004), and surCTLA-4+CD8+ lymphocytes (0.56 ± 0.53 and 0.26 ± 0.27 respectively, P = 0.002) (Table 2).

## Discussion

CTLA-4 is significant for tumors to evade host immune surveillance, and has been implicated in immune dysregulation of lung cancer^28^, cervical cancer^29^, breast cancer^30^, skin cancer^31^, gastric cancer^32^, colorectal cancer^33^, B cell chronic lymphocytic leukemia and non-Hodgkin’s lymphoma^34^. However, the prognostic role and clinical application of CTLA-4 in tumors are still controversial, which might due to the differences in experiential methods and study populations. For example, non-small cell lung cancers (NSCLC) with CTLA4 overexpression were associated with a reduced death rate^16^. Higher clinical stage and promoted axillary lymph node metastasis can be found in breast cancer patients with higher CTLA-4 mRNA levels^35^. CTLA4 downregulation led to a significant increase in the proliferation and survival of chronic lymphocytic leukemia cells^1^. However, little evidence showed correlations between CTLA-4 and prognosis in cancer patient. Thus, our meta-analysis aimed to illustrate the prognostic role of CTLA-4 expression in several of cancers. CTLA-4 have been discovered to be expressed in B lymphocytes^36^, CD4 and CD8 subsets of T cells and monocytes^37^,^38^, which could be wildly detected in tumors, lymph nodes, and spleen. However, CTLA-4 could not be detected in a series of non-lymphoid tissues. CTLA-4 consists of two isoforms: a full-length membrane-bound receptor isoform and sCTLA-4, which consists of only the extracellular domain^7^,^39^. Studies indicated that the expression of CTLA-4 isoforms was at the same level in CD4 cells, while CD8 cells appear to express nearly 2.5-fold more full-length product with respect to sCTLA-4^40^.

To our knowledge, this is the first systematic review and meta-analysis to explore and report the prognosis value of CTLA-4 in various cancers. The pooled HR of 14 eligible studies for OS in our meta-analysis concluded that no significant correlation was showed between CTLA-4 expression and OS in tumors (HR: 1.24, 95% CI: 0.86–1.56). This result didn’t in accordance with the previous findings that CTLA-4 might be a favorable/negative indicator of cancers on OS, which may due to the controversy and complexity of the function, expression, and location in different isoforms of CTLA-4. Nevertheless, our analysis indicated that sCTLA-4 subgroup, analyzing by ELISA, has a strong tendency to be associated with good prognosis in malignant tumor, though no significance was showed in this article (HR: 0.63, 95% CI: 0.37–1.08). While the cytoplasmic tail is shorter than that of the full-length form of CTLA-4^41^,^42^,^43^, sCTLA-4 contains the extracellular MYPPPY motif which plays a crucial role in binding B7 molecule^44^. Thereby, sCTLA-4 maintains the function of binding CD80/CD86 natural ligands expressed on antigen-presenting cells (APCs). Nevertheless, the immunoregulatory functions of sCTLA-4 seem to be quite complicated. On one hand, sCTLA-4 is capable to bind CD80/CD86 and participate in the inhibitory regulation pathway to suppress T cell activation, which is similar with the full-length CTLA-4^45^. On the other hand, lacking the transmembrane domain disabled sCTLA-4 in downregulating immune function; thereby block the negative signal of CTLA-4 after interaction with CD80/CD86 in the later phases of T-cell responses. Consequently, the inconsistency effect of sCTLA-4 potentiates it to be correlated with favorable prognosis. The complex regulatory role of sCTLA-4 could also act at cytokine levels. IFN-γ, IL-2, and -13 productions were observed to be sharply reduced following B7/CTLA-4/CD28 cross-linkage, while IL-10 and TGF-β increased. All cytokine changes could participate in the regulation of cell-mediated autoimmunity^46^,^47^. Besides, several studies indicated that the expression of sCTLA-4 is associated with proinflammatory cytokine levels. Grohmann *et al*. found that sCTLA-4 expression can be enhanced by interferon beta-1a (IFN-β1a) in mononuclear cells of healthy individuals^48^. The research showed no significant difference in the intensity analysis of full-length CTLA-4 from incubated mononuclear cells, whereas the cDNA intensity of sCTLA-4 was significantly enhanced following IFN-β1a stimulation. This result verified the immunomodulatory effects of sCTLA-4 by IFN-β1a and the influence are selectively^48^. In addition, it has been shown that sCTLA-4, as well as the recombinant sCTLA-4 protein (CTLA4-Ig), is capable to suppress the mixed leukocyte reaction in dose-dependent manners^36^,^45^,^49^. Studies^50^,^51^ indicated that CTLA4-Ig plays crucial roles in T-cell inhibition via inducing different types of APCs including dendritic cells^52^. The correlation of sCTLA-4 and proinflammatory cytokine levels also certified the immunoregulatory capability of sCTLA-4 *in vivo*^53^.

Subsequently, more subgroup analyses were conducted to further investigate the prognostic role of CTLA-4. Interestingly, the pooled HR for OS was 1.47 (95% CI: 1.14–1.89) in SNP subgroup, which suggested that CTLA-4 +49AA genotype was an independent adverse indicator for cancer prognosis. The CTLA-4 gene has more than one hundred SNPs, among which the most studied phenotypes was +49 A/G located in exon 1, and CT60 AA genotype, located in the 30-untranslated region of the gene^54^. The CTLA-4 +49AA genotype was identified in this study to be an independent adverse prognostic indicator in cancer patients. In the meanwhile, patients with CTLA-4 +49AA genotype had significantly shorter survival time than those with the GG or GA genotype. The effect of CTLA-4 +49AA genotype on the development, progression and prognosis of cancers may due to the interference with the transcriptional activity of CTLA-4 mRNA level^55^. The 49AA genotype is associated with increased expression of CTLA-4 mRNA and protein, which has enhanced interaction with B7 ligands and an enhanced effect on T cell inhibition^55^,^56^. In addition, compared with other genotypes, patients with the CTLA-4 +49AA genotype had significantly increased CTLA-4 expression on peripheral blood mononuclear cells, and decreased interleukin-2 expression which is crucial in T cell growth^57^. Thus, tumor patients with CTLA-4 +49AA genotype would be expected to have an enhanced suppression on T cell activity and immune function, which contribute to the poor prognosis of cancers.

The subgroups categorized by cancer types showed significant correlations between CTLA-4 and OS in nasopharyngeal carcinoma, esophageal carcinoma, malignant hematologic diseases, and glioblastoma, most of which had a strong pooled HR. In addition, we observed a favorable effect of CTLA-4 overexpression on OS in malignant pleural mesothelioma, which may due to the complexity of CTLA-4 functions. Salvi *et al*.^16^ suggested that CTLA-4 could mediate inhibitory function on tumor cells, comparable with its suppression effect for T cells. Recent studies showed that early disseminated NSCLC cells could produce CTLA-4 and subsequently suppress proliferation and/or enhance apoptosis of cancer cells via engagement with B7 ligands^58^,^59^,^60^. In this regard, CTLA-4 was associated with good clinical outcome and longer survival due to its direct antiproliferative and proapoptotic effects in cancers. However, Huang *et al*.^5^ confirmed that the low tumor CTLA-4 expression group has a significant longer OS, failure-free survival (FFS), and distant failure-free survival (D-FFS) compared to those groups with high tumor CTLA-4 expression. A Cox regression analysis confirmed the prognostic value of the tumor CTLA-4 expression especially for the D-FFS of nasopharyngeal carcinoma patients. This result didn’t in accordance with the previous findings that CTLA-4 might be a favorable indicator of cancers on OS. Notably, the subgroups of lung cancer and breast cancer showed no significance, the aforementioned wildly expression, contradictory effects on tumor and T cell activity, different types of isoforms, as well as limited include studies and publication bias all contribute to the negative outcome.

Our analysis also observed increased expression of sur- and in-CTLA-4 expressing lymphocytes in cancer patients. Researches indicated that intracellular CTLA-4 consists of the majority of molecules present of lymphocytes, while only less than 1% expressed on the surface (surCTLA-4). This observation is in consistent with the dynamic of CTLA-4 expression. After lymphocytes being antigen-specific activated, CTLA-4 expressed on the surface is rapidly internalized intracellular compartment of lymphocytes by clathrin-dependent endocytosis^61^. After reactivation, intracellular CTLA-4 transferred to the surface and exhibited its inhibitory effect of T cells, while the internalization could be inhibited via the formation and co-internalization of TCR complex^62^,^63^. Several of studies have notified the correlation between CTLA-4 lymphocyte variants and its susceptibility to autoimmunity and cancer^64^. Erfani *et al*. indicated that surCTLA-4 in lymphocyte subsets of patients with laryngeal squamous cell carcinoma or non-small cell lung cancer was higher in comparison to healthy controls^65^,^66^. The differences were significant in inCTLA-4+CD4+ lymphocytes, surCTLA-4+CD4+ lymphocytes, inCTLA-4+CD8+ lymphocytes, and surCTLA-44+CD8+ lymphocytes. However, the results also have restrictions which may due to the limited studies associated with intercellular CTLA4 and/or that present on the surface or of lymphocyte.

Recently, soluble intact anti-CTLA-4 antibody which is capable to enhance antitumor immunity via greatly boosting T-cell responses to both antigen and superantigen, has become a hotspot^58^,^62^. The antitumor effects had been wildly verified in murine model of fibosarcoma^67^, colon carcinoma, and metastatic melanoma models^68^. All the animal studies confirmed the safety of blocking antibodies, and provided proof that CTLA-4 inhibition could lead to potent antitumor effects in cancer patients^67^,^69^. Notably, the sensitivity of poorly immunogenic tumors to anti-CTLA-4 antibodies was low, whereas this condition for animals could be promoted by treatment with cellular vaccines transduced by granulocyte-macrophage colony-stimulating factor (GM-CSF)^69^. These observations promoted several of clinical trials for fully human anti-CTLA-4 monoclonal antibodys (mAbs) including tremelimumab and ipilimumab. However, a Phase III trial concerning tremelimumab was halted due to the limited therapeutic benefit compared with traditional chemotherapy, though it might still be valuable when used as adjuvant combination therapy^70^,^71^. Ipilimumab was approved in 2011 as antitumor therapy for metastatic melanoma patients, while the treatment is still evaluated for NSCLC, prostate, and pancreatic cancers, among others. Recent trials showed that 22–25% of patients responsive to ipilimumab achieved long-term survives beyond 3 years, among whom some suffering from active disease reached for extended periods 5 years^72^. Recently, it is a hot spot in evaluating the therapy of anti-CTLA-4 mAb combined with immunostimulatory agent, including anti-PD-1 mAb^73^, GM-CSF vaccination^74^, and immune stimulatory cytokines including IFN-α2b^75^ and IL-15^76^. Thus, there is no doubt that anti-CTLA-4 mAb therapy, as well as in combination with other immune-activating agents, could offer a significant improvement in antitumor immune effect in cancer patients.

Cut-off values adopted are varies among different patient cohorts that would result in bias. In these studies, median and/or mean values are mostly used in eligible articles. The data extrapolation methods were also correlated with publication bias. In addition, not all articles reported results of multivariate survival analysis which were considered the most reliable data. We collected all the relevant researches through quantities of databases and different search strategies to minimize this bias. However, more of less literature could have been missed during the collection and the publication bias is inevitably. We assessed publication bias in articles correlated with OS for all eligible studies and reported negative, thus the result was acceptable despite publication bias.

This meta-analysis has several limitations. Firstly, rather than individual record which is considered most reliably by some authors, the data in our article was obtained from published literatures^77^. Secondly, our meta-analysis only consists of articles published in English to facilitate understanding. Since the negative results always published in native language, this strategy could benefit the articles with positive conclusion^78^. Furthermore, the number of patients included in subgroups was relatively small, though the whole population is considerably, which could led to the restricted value of this meta-analysis.

In conclusion, the meta-analysis indicated that no significance was found when analyzing the overall effect of CTLA-4 expression on OS in several of cancer cases. Nevertheless, CTLA-4 +49AA genotype was an independent adverse indicator for cancer prognosis, while the high-expression of sCTLA-4 has a tendency to be correlated with the prolonged OS.

## Additional Information

**How to cite this article**: Hu, P. *et al*. The prognostic value of cytotoxic T-lymphocyte antigen 4 in cancers: a systematic review and meta-analysis. *Sci. Rep.* **7**, 42913; doi: 10.1038/srep42913 (2017).

**Publisher's note:** Springer Nature remains neutral with regard to jurisdictional claims in published maps and institutional affiliations.

## Figures and Tables

**Table 1:** Main characteristics of eligible studies.

Author	Year	Country	N	Cancer	Stage	Adjuvant therapy	Source	Method	Cut-off criteria	Cut-off value	Analyze method	HR	Survival analysis
Huang *et al*.	2016	China	191	NPC	I–IV	RT, CCRT	tumor, LYM	IHC	median	H-score of 0.7	MVA	report	OS
Roncella *et al*.	2016	Italy	45	MPM	I–IV	NM	blood, tumor	IHC, ELISA	median	S->66 PE->67	MVA	report	OS
Fukuda *et al*.	2016	Japan	181	renal cancer	I–IV	targeted therapy	blood	ELISA	NM	NM	UA	DE	OS
Zhang *et al*.	2015	China	158	esophageal cancer	I–IV	NM	tumor	IHC	mean	H-score of 2–4	MVA	report	OS
Yu *et al*.	2015	China	130	breast cancer	I–IIIC	RT, CT, ET	tumor; LYM	IHC	ROC curve	H-score of 1.525	MVA	report	OS
Teng *et al*.	2015	China	62	rectal cancer	III–IV	CCRT	Tumor	IHC	NM	H score of 20	UA	Report	OS
Sengsayadeth *et al*.	2014	America	780	hematological cancer	NM	HSCT	SNP	PCR	NM	NM	MVA	K-M	OS
Fong *et al*.	2013	America	24	glioblastoma	NM	immunotherapy	LYM	FC	RPA	1.047:0.8065	UA	report	OS
Salvi *et al*.	2012	Italy	81	LC	I–IIIB	None	tumor	IHC	median	H-score of 20	MVA	report	OS
Jagasia *et al*.	2012	America	164	hematological cancer	NM	HSCT	SNP	PCR	NM	NM	MVA	report	OS
Wang *et al*.	2012	UK	284	melanoma	II–III	IFN-a Therapy	tumor	PCR	median	NM	MVA	report	OS
Xiao *et al*.	2012	China	240	hematological cancer	NM	immunotherapy	SNP	PCR	NM	NM	MVA	K-M	OS
Song *et al*.	2011	China	338	LC	IIIB–IV	RT, CT, CRT	SNP	PCR	N/A	N/A	MVA	report	OS
Pe´rez-Garcı´a *et al*.	2009	Spain	143	AML	NM	CT	SNP	PCR	NM	NM	MVA	Report	OS
Erfani *et al*.	2013	Iran	72	laryngeal cancer	I–IV	None	LYM	FCM	N/A	N/A	N/A	N/A	N/A
Erfani *et al*.	2012	Iran	39	LC	II–IV	None	LYM	FCM	N/A	N/A	N/A	N/A	N/A

N, number; OS, overall survival; HR, hazard ratio; MVA, multivariate analysis; UA, univariate analysis; PCR, polymerase chain reaction; IHC, immunohistochemistry; N/A, not applicable; H score, histochemical score; RPA, recursive partitioning analysis; NPC, nasopharyngeal cancer; MPM, malignant pleural mesothelioma; LC, lung cancer; AML, acute myelocytic leukemia; RT, radiotherapy; CT, chemotherapy; CCRT, concurrent chemoradiotherapy; ET, endocrine therapy; HSCT, hematopoietic stem cell transplantation; SNP, single nucleotide polymorphisms; LYM, lymphocyte; FCM, flow cytometry; DE, data extrapolated.

**Table 2:** Mean percentage of CTLA4+ lymphocyte subsets in cancer patients and healthy donors.

	Patients			Groups			P-value
	N	mean	SD	N	mean	SD	
Surface CTLA4 lymphocytes							
Sur-CD4+	58	0.78	0.65	39	0.54	0.51	0.000
Sur-CD8+	58	0.56	0.53	39	0.26	0.27	0.002
Sur-CD19+	56	0.26	0.30	32	0.17	0.18	0.109
Intra cellular CTLA4 lymphocytes							
In-CD4+	58	8.66	7.49	39	4.17	6.11	0.002
In-CD8+	57	9.12	9.72	36	3.5	7.65	0.004
In-CD19+	58	0.89	1.98	33	0.3	0.35	0.095

Sur-, surface CTLA-4; In-, intra CTLA-4; N, number; SD, standard deviation.
